# Characterisation and comparison of adipose tissue macrophages from human subcutaneous, visceral and perivascular adipose tissue

**DOI:** 10.1186/s12967-016-0962-1

**Published:** 2016-07-11

**Authors:** Ivana Kralova Lesna, Anna Kralova, Sona Cejkova, Jiri Fronek, Marek Petras, Alena Sekerkova, Filip Thieme, Libor Janousek, Rudolf Poledne

**Affiliations:** Laboratory for Atherosclerosis Research, Centre for Experimental Medicine, Institute for Clinical and Experimental Medicine, Prague, Czech Republic; Transplant Surgery Department, Institute for Clinical and Experimental Medicine, Prague, Czech Republic; Department of Clinical and Transplant Immunology, Institute for Clinical and Experimental Medicine, Prague, Czech Republic; 2nd Faculty of Medicine, Charles University in Prague, Prague, Czech Republic

**Keywords:** Adipose tissue, Macrophages, Inflammation, Menopause

## Abstract

**Background and aims:**

Macrophages play important roles in adipose tissue inflammation and its consequences. Unfortunately, a detailed description of the macrophage phenotypes in different human adipose tissues is not available.

**Subjects and methods:**

Subcutaneous, visceral and perivascular adipose tissues were obtained from 52 living kidney donors during live donor nephrectomy. Stromal vascular fractions were isolated, and the macrophage phenotypes were analyzed by flow cytometry using surface markers (CD14, CD16, CD36, and CD163).

**Results:**

In addition to CD16 positivity, pro-inflammatory macrophages also display high scavenger receptor CD36 expression. The great majority of CD16 negative macrophages express the anti-inflammatory CD163 marker. The presence of pro-inflammatory macrophages was almost twice as high in visceral (p < 0.0001) and perivascular (p < 0.0001) adipose tissues than in subcutaneous tissue. This difference was substantially more pronounced in the postmenopausal women subgroup, consequentlly, the total difference was driven by this subgroup.

**Conclusion:**

We obtained detailed information about M1 and M2 macrophage phenotypes in human adipose tissue. The visceral and perivascular adipose tissues had substantially higher pro-inflammatory characteristics than the subcutaneous tissue. The higher proportion of pro-inflammatory macrophages in the visceral adipose tissue of postmenopausal women might be related to an increased cardiovascular risk.

## Background

Recently, interest in the field of adipose tissue biology has been increasing. The principal focus has been on subclinical low-grade inflammation, which has been suggested to link obesity to its metabolic complications, especially insulin resistance and type 2 diabetes mellitus [1]. Adipose tissue was considered a rather inert organ for many years and was viewed as an energy store that could be mobilized during periods of starvation. In the 1990s, this approach was completely revised following the discovery of the massive endocrinal, paracrine, and autocrine potential of adipose tissue [2]. In addition to its role in energy metabolism, adipose tissue may impact both local and systemic metabolic processes during healthy and unhealthy periods [3].

Chemoattractive cytokines influence macrophage trafficking. There is considerable evidence from animal and human experiments that obese adipose tissue is markedly infiltrated by macrophages [4–7]. At the tissue level, inflammatory pathways are connected to quantitative and phenotypic changes in adipose tissue macrophages (ATM); these cells appear to be at the center of obesity-related inflammation [8]. Once recruited, the macrophages themselves secrete additional chemokines, which initiate a feed-forward loop that potentiates the inflammatory response [9]. Macrophage infiltration and pro-inflammatory gene expression in adipose tissue together with the increase in subclinical inflammatory markers precede the metabolic consequences [5]. Therefore, macrophages may be important players in the initiation of insulin resistance. Macrophage turnover in tissues is the result of several biological processes, including recruitment, local proliferation, apoptosis, and macrophage egress [10, 11].

Macrophage populations are highly heterogeneous, and several activation states of macrophages have been observed. This macrophage diversity has led to an effort to categorize the macrophage populations. The most prevalent approach is to define the M1 and M2 systems primarily by macrophage properties in vitro. M1 macrophages are generated by treating monocytes with lipopolysaccharide or interferon-γ, whereas increasing concentrations of IL-4/IL-13 or IL-10 are inducers of M2 macrophages [12, 13]. Although these in vitro studies are demonstrative, an in vivo analogy can only be hypothesized. M1 macrophages are predominant in sites of inflammation and therefore are considered to be pro-inflammatory primarily due to their high production of pro-inflammatory cytokines. Both the numbers and proportions of these two phenotypes might play important roles [8]. However, a strict definition of adequate M1 and M2 macrophage surface markers has not been clear to date.

Obesity is also associated with qualitative changes in ATM phenotypes and functions [14]. An animal model showed that external stimuli, such as physical activity or diet, could induce a switch in macrophage subpopulations [15–17]. However, because macrophage markers differ between rodents and humans, it is difficult to extrapolate these results to human adipose tissue. Additionally, results from flow cytometry analyses of human adipose tissue [4, 18, 19] suggest that an individual ATM might simultaneously bear both M1 and M2 characteristics [4]. Adipose tissues isolated from different depots demonstrate different features [20]. Thus, it seems probable that the differences in non-adipose cell types may contribute to this finding. The aims of our study were as follows:

- To determine the macrophage subpopulations in human adipose tissue.
- To compare the proportions of the macrophage subpopulations in the subcutaneous (SCAT), visceral (VAT), and perivascular (PVAT) tissues of healthy individuals.
- To determine the possible relationship of the above-mentioned data with age.

We utilized the recently enlarged transplantation program of living kidney donors at our institute to obtain fresh, preoperatively isolated human adipose tissues.

## Methods

### Study participants

SCAT, VAT (outside of Gerota’s fascia), and PVAT (surrounding the arteria renalis) were intra-operatively obtained from living kidney donors during hand-assisted retroperitoneoscopic live donor nephrectomy. Apart from the SCAT, the removal of VAT and PVAT is a standard procedure prior to transplantation.

Clinical data were collected from clinical documentation of the enrolled subjects and from an interview targeting lifestyle factors. Prior to enrollment in the study, the subjects were thoroughly informed about the study and informed consent forms were signed. The study was approved by the local Ethics Committee.

### Biochemistry

The total cholesterol, triglyceride and HDL cholesterol fractions were determined from fasting blood samples obtained immediately prior to the operation (prior to anesthesia) using an enzymatic method (Hoffmann-LaRoche, Switzerland). The concentration of the HDL fraction was analyzed after the precipitation of apoprotein B-containing particles using a phosphotungstate method. hsCRP (high-sensitivity C-reactive protein) was measured using an immunoturbidimetric assay with an autoanalyzer (Cobas Mira Plus, Roche, Basel, Switzerland).

### Stromal vascular fraction isolation

The stromal vascular fraction (SVF) was separated using a procedure modified according to Zuk [21]. Approximately 2-gram SCAT, VAT and PVAT samples (surrounding the arteria renalis) were cleaned of connective tissue, blood vessels, and blood residue. The samples were minced with scissors (approximately 1 mm^3^) and exposed to collagenase (2 mg/ml, Sigma-Aldrich) in phosphate-buffered saline solution for 20 min at 37 °C. The digestate was immediately cooled and subsequently filtered using two filters (150 μm and then 50 μm). After repeated washing, the stromal vascular fraction was obtained.

### Macrophage markers

Based on data from the literature [18, 20, 22–24] and our recent results [25], we suggest that macrophages with high phagocytic activity characterized by CD16 expression and high CD36 expression but no CD163 expression should correspond to normally stimulated M1 macrophages. Conversely, macrophages with no CD16 expression but CD163 positivity might be considered anti-inflammatory M2 macrophages. We are aware that this classification could oversimplify the in vivo situation where the full phenotypic spectrum of transient phenotypes between M1 and M2 may exist.

### Flow cytometry analysis

Stromal vascular cells and blood samples from the subjects were incubated with a cocktail of monoclonal antibodies conjugated with specific fluorochromes [CD14-phycoerythrin (PE)-cyanine 7, CD16-phycoerythrin-Texas Red-X, CD36-fluorescein isothiocyanate (FITC), and CD163-PE clone RM3/1] and the appropriate isotype controls (Beckman Coulter) for 30 min shielded from light. All of the antibodies were purchased from Beckman Coulter (Brea, CA, USA) except for CD163 (BioLegend, CA, USA). The flow cytometry analysis was performed within 2 h of staining.

All flow cytometry analyses were performed on a CyAn ADP 9C flow cytometer using the Kaluza software (Beckman Coulter, Brea, CA, USA).

**Fig. 1:**
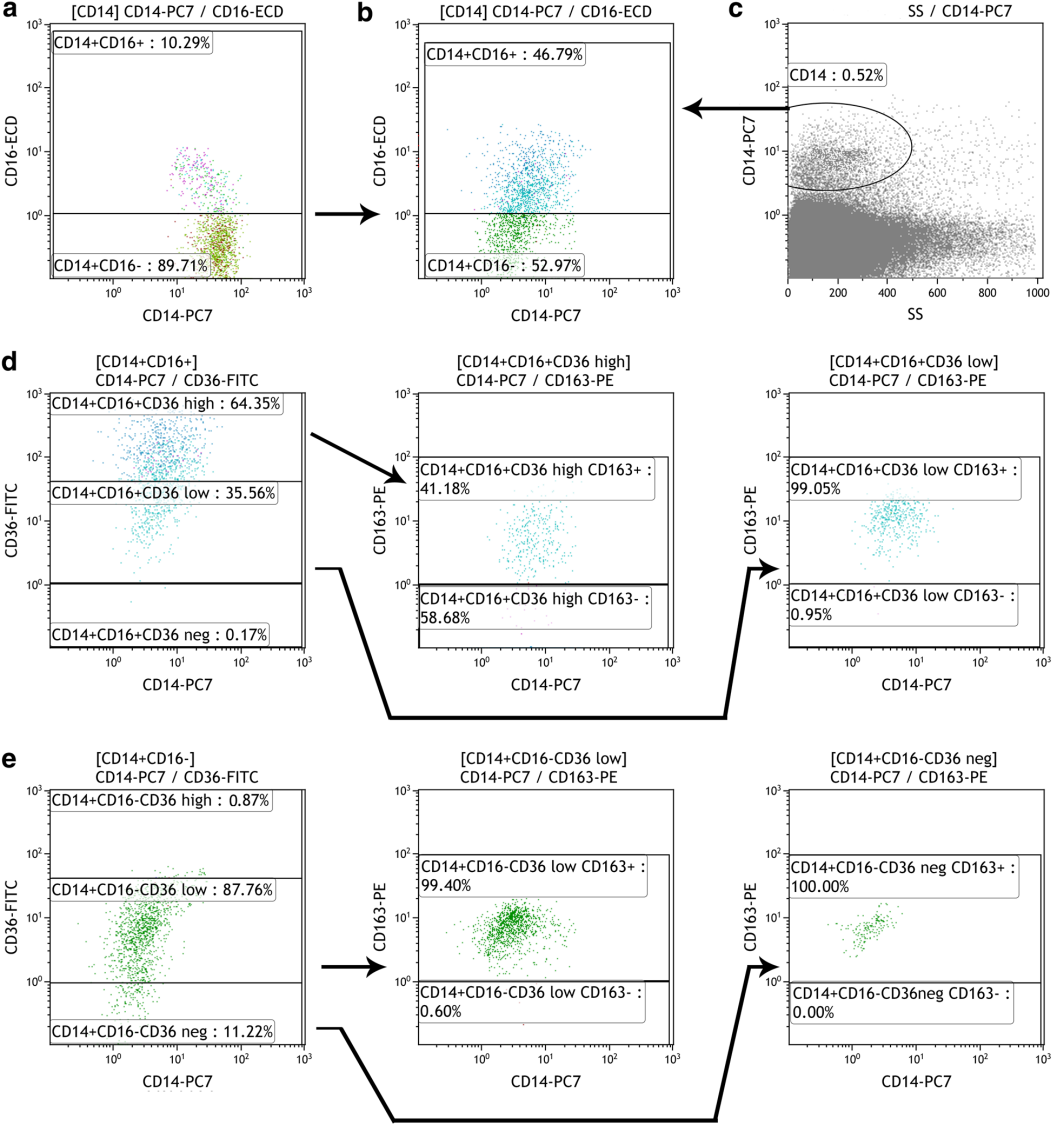
Example of the SVF flow cytometric analysis. **a** CD16 positive monocytes were first identified and delineated in the blood sample (*left*, CD16positive macrophages in the *upper part*). The setting was fixed and subsequently used for the SVF analysis (**b**). Total macrophages in the SVF were identified by positivity for CD14 (**c**). Based on the CD16 marker, two subpopulations were distinguished (**b**, CD16-positive macrophages in the *upper part*). **d** The CD16+ subpopulation was divided according to CD36 marker markers (**d**, *left*) and then the CD163 presence was determined within both the CD36 positive subpopulations [i.e., CD16+ CD36^high^ (**d**, *middle*) and CD16+ CD36^low^ (**d**, *right*)]. Similarly, the CD16 negative subpopulation was divided according to the CD36 marker (**e**, *left*) and then the CD163 presence was determined in both the CD16− CD36^low^ (**e**, *middle*) and CD16− CD36− subpopulations (**e**, *right*)

Due to difficulties in delineating CD16 positive cells in the SVF, the CD16 positive monocytes were first identified and delineated in a blood sample where the CD16 positive subpopulation was clearly visible. The setting was fixed and subsequently used for the SVF analysis. The gating strategy used to identify the SVF macrophage subpopulations is shown in Fig. 1.

The proportion of ATM subpopulations was expressed as a percentage of all monocytes/macrophages. The absolute number of macrophages per gram of adipose tissue was determined by calculating the dilution and the amount of adipose tissue applied. Additionally, the proportion of ATM subpopulations was expressed as a percentage of all macrophages. Due to technical difficulties, some samples were not analyzed for absolute numbers of monocytes (approximately 5 % of the adipose tissue samples).

The viability of the analyzed cells was measured for each sample using the 7-aminoactinomycin D (7-AAD) method. Only samples with viabilities higher than 75 % were considered.

Subpopulations representing less than 5 % were omitted from the analysis.

Because the main fraction of blood monocytes (CD14+ CD16−163−) was not observed in the tested adipose tissue samples, the confounding effect of blood contamination (despite thorough repeated washing of the samples) can be neglected.

### Statistical analysis

The investigated subpopulations were normally distributed. Therefore, the significance of the differences between them was tested using the appropriate unpaired or paired Student’s t test method. All tests were two tailed, and the level of significance was set at 0.05. The statistical analyses and correlation were performed with the biostatistics software Prism, version 5 (GraphPad Prism).

## Results

A total of 52 living kidney donors were identified (19 men and 33 women, out of whom 16 were premenopausal and 17 were postmenopausal ages). However, eight subjects did not agree with SCAT removal and therefore only 44 SCAT samples were analyzed. Our study group was generally healthier than a gender- and age-matched group selected from a large representative sample of the same population [13] and therefore could be regarded as relatively healthy individuals. However, due to the recently more flexible criteria for living kidney donation, the prevalence of light hypertension was approximately 20 %, overweight was 36 %, obesity was 13 and 33 % of the subjects were smokers. The increased LDL concentration prevalence was 8 %, the decreased HDL concentration (lower than 1.0 mmol l^−1^ in men and 1.3 mmol l^−1^ in women) was 58 % and hypertriglyceridemia (more than 1.6 mmol l^−1^) was present in 26 % of subjects.

No differences in age were found in the men compared to the women (45.09 ± 8.98 and 46.60 ± 11.53 years) (Table 1). The mean BMI of the men was 26.94 ± 3.74 kg/m^2^ and the mean BMI of all women was 25.17 ± 3.48 kg/m^2^ (p = 0.05); there was no difference in BMI between women with pre- and postmenopausal ages. The total cholesterol concentrations of the men did not differ from the women. Nevertheless, the older women had substantially higher total cholesterol than the younger women (p < 0.05). No differences in non-HDL cholesterol values were found in the men compared to the women, with higher non-HDL cholesterol concentration in the women of postmenopausal age than in the women of premenopausal age (p < 0.02). The male group displayed significantly lower HDL concentrations compared to all of the women (p < 0.01); no differences were observed in the female subgroups. No differences were observed in the mean fasting triglyceride concentrations in men compared to women and between women with pre- and post-menopausal ages. There were no gender or age differences in CRP between the groups.

**Table 1:** Characteristics of the living kidney donor subgroups

Parameters	Women (W)	Men (M)	Significance (M × W)	PreW	PostW	Significance (PreW × PostW)
Age (years)	46.60 ± 11.53	45.09 ± 8.98	n.s.	43.22 ± 5.26	56.24 ± 5.48	p < 0.0001
BMI (kg m^−2^)	25.17 ± 3.48	26.94 ± 3.74	p = 0.05	24.42 ± 3.39	25.97 ± 3.49	n.s.
Total cholesterol (mmol l^−1^)	4.37 ± 1.01	4.32 ± 0.64	n.s.	4.01 ± 0.70	4.86 ± 1.20	p < 0.05
HDL-C (mmol l^−1^)	1.32 ± 0.35	0.99 ± 0.26	p < 0.01	1.32 ± 0.26	1.31 ± 0.47	n.s.
nonHDL-C (mmol l^−1^)	3.15 ± 1.07	3.39 ± 0.64	n.s.	2.69 ± 0.68	3.54 ± 0.99	p < 0.02
Triglycerides (mmol l^−1^)	1.23 ± 0.78	1.57 ± 0.64	n.s.	1.11 ± 0.42	1.39 ± 1.10	n.s.
hsCRP (mg l^−1^)	1.66 ± 2.90	1.18 ± 1.34	n.s.	1.61 ± 2.33	1.72 ± 3.67	n.s.

Data are expressed as the mean of the proportion ±SD and by significance according to the Student’s unpaired parametric t test

*W* Women (n = 33), *M* men (n = 19), *PreW* premenopausal women (n = 16) and *PostW* postmenopausal women (n = 17), *n.s*. non-significant

The total number of macrophages per gram did not significantly differ between the SCAT, VAT and PVAT (10,900 ± 12,250, 13,200 ± 10,350 and 15,650 ± 15,100 macrophages/g, respectively). When the SVF macrophages were divided according to CD16 positivity, a lower proportion of CD16 positive macrophages was found in the SCAT compared to the VAT (48.9 ± 14.3 vs. 53.4 ± 13.3 %; p < 0.02) (Table 2).

**Table 2:** Different phenotypes of macrophages isolated from adipose tissues

CD14+				SCAT	Significance (SCAT × VAT)	VAT	Significance (VAT × PVAT)	PVAT	Significance (SCAT × PVAT)
CD16	CD36	CD163							
+			%	48.9 ± 14.3	p < 0.02	53.4 ± 13.3	n.s.	50.6 ± 14.0	n.s.
+	+++		%	32.7 ± 13.7	p < 0.02	39.4 ± 13.4	n.s.	38.7 ± 15.5	p < 0.05
+	+		%	14.1 ± 7.7	n.s.	12.4 ± 7.5	n.s.	10.4 ± 6.9	p < 0.01
+	+++	+	%	19.9 ± 9.5	n.s.	15.4 ± 6.8	n.s.	13.5 ± 8.1	p < 0.005
+	+++	−	%	14.3 ± 8.0	p < 0.0001	25.5 ± 11.8	p < 0.05	29.2 ± 12.6	p < 0.0001
+	+	+	%	13.1 ± 6.2	n.s.	11.8 ± 6.8	p < 0.05	9.4 ± 5.3	p < 0.005
−			%	51.1 ± 14.3	p < 0.02	46.6 ± 13.3	n.s.	49.4 ± 14.0	n.s.
−	+		%	45.0 ± 13.5	p < 0.02	40.4 ± 12.1	n.s.	41.8 ± 12.3	n.s.
−	−		%	6.1 ± 5.0	n.s.	6.9 ± 6.3	n.s.	7.6 ± 7.2	n.s.
−	+	+	%	43.9 ± 13.1	p < 0.02	39.0 ± 12.2	n.s.	39.7 ± 12.5	n.s.
−	−	+	%	5.8 ± 4.9	n.s.	5.8 ± 6.2	n.s.	7.1 ± 6.9	n.s.

Proportions of macrophages isolated from subcutaneous (SCAT, n = 44), visceral (VAT, n = 52) and perivascular (PVAT, n = 52) adipose tissues, *n.s*. non-significant

Results are expressed as the mean of the proportion ±SD and by significance according to the Student’s paired parametric t test

In all tissues, CD16+ macrophages were also CD36 positive, and two subpopulations could be clearly distinguished (CD36^low^ and CD36^high^). The proportion of CD16+ CD36^high^ macrophages was higher in the VAT and PVAT (39.4 ± 13.4 and 38.7 ± 15.5) than in the SCAT (32.7 ± 13.7 %, p < 0.02 and p < 0.05, respectively); no difference was observed between the VAT and PVAT.

When these CD16+ CD36^high^ macrophages were divided according to the presence of CD163, the SCAT macrophages were approximately evenly distributed (19.9 ± 9.5 % CD163+ and 14.3 ± 8.0 % CD163−), whereas the proportion of CD163+ macrophages accounted for only half of the CD163-negative macrophages in the VAT (15.4 ± 6.8 and 25.5 ± 11.8 %) and PVAT (13.5 ± 8.1 and 29.2 ± 12.6 %). The only significant difference in the CD16+36^high^163+ subpopulation was between the SCAT and PVAT (p < 0.005). Conversely, the differences between the SCAT and both the VAT and PVAT were highly significant when the CD16+36^high^ CD163− subpopulation that corresponded to pro-inflammatory M1 macrophages was analyzed (both p < 0.0001).

The CD16+36^low^ macrophage prevalence was higher in the SCAT than in the PVAT (p < 0.01), but no other significant differences between the adipose tissues were observed. In this subpopulation, the majority of cells (~90 %) also expressed the CD163 surface marker. When the presence of the CD163 marker was included, the CD14+ CD16+36^low^163+ subpopulation was significantly lower in both the visceral (p < 0.05) and perivascular (p < 0.005) adipose tissues compared to the subcutaneous tissue.

In contrast to the CD16+ macrophages, macrophages lacking CD16 exhibited low CD36 expression. However, only a minor proportion of this subpopulation (approximately 7 %) did not express the CD36 marker at all.

The majority of the CD16− CD36^low^ macrophages were equivocally CD163− positive, as were the majority of the CD16− CD36− macrophages. Therefore, when all of the CD16− macrophages were evaluated together, 95 ± 4 % expressed the anti-inflammatory marker CD163. Consequently, the proportions of CD16− CD36^low^ and CD16− CD36^low^ CD163+ macrophages were also higher in the SCAT compared to the VAT (p < 0.02).

In the whole group of living kidney donors, the proportion of pro-inflammatory macrophages was lower in the SCAT compared to the VAT and PVAT. These differences increased when their phenotypes became gradually more specified from CD16+ to CD16+ CD36^high^ and finally to CD16+36^high^ CD163−.

Upon analyzing the data from a relatively large set of SVFs obtained from different adipose tissues, we found substantial differences between the subgroups of women with premenopausal (n = 16) and postmenopausal (n = 17) ages (Table 3). No substantial differences were found when the males were compared with the entire group of women. Therefore, we compared selected ATM subpopulations according to their menopausal status. The cut-off point for the menopausal status was 51 years, and the menopausal status was verified using a follicle-stimulating hormone measurement (FSH > 29 IU/ml). Although no significant differences in the proportion of CD16+ subpopulations were found in the different adipose tissues of women of premenopausal age (Table 3), a highly significant difference was found between the SCAT and VAT (p < 0.005) in the subgroup of women of postmenopausal age in the presence of pro-inflammatory macrophages. Women of postmenopausal age also had a higher proportion of CD16+ macrophages in the VAT compared to women of premenopausal age (59.0 ± 12.0 % vs. 47.2 ± 15.1, p < 0.02).

**Table 3:** Different phenotypes of macrophages isolated from adipose tissues from women

CD14+				Women	SCAT	Significance (SCAT × VAT)	VAT	Significance (VAT × PVAT)	PVAT	Significance (SCAT × PVAT)
CD16	CD36	CD163								
+			%	PreW	47.8 ± 13.9	n.s.	47.2 ± 15.1	n.s.	48.1 ± 12.7	n.s.
				PostW	47.8 ± 14.6	p < 0.005	59.0 ± 12.0	n.s.	55.7 ± 13.7	n.s.
				P* (PreW × PostW)	n.s.		p < 0.02		n.s.	
+	+++		%	PreW	30.8 ± 13.4	n.s.	34.7 ± 16.0	n.s.	34.8 ± 13.0	n.s.
				PostW	31.7 ± 14.7	p < 0.0005	42.8 ± 11.9	n.s.	45.2 ± 15.3	p < 0.001
				P* (PreW × PostW)	n.s.		n.s.		p < 0.05	
+	+++	−	%	PreW	13.7 ± 8.9	p < 0.02	22.0 ± 6.3	n.s.	25.2 ± 12.5	p < 0.005
				PostW	16.3 ± 8.8	p < 0.0005	28.0 ± 11.4	p < 0.05	32.3 ± 11.4	p < 0.0001
				P* (PreW × PostW)	n.s.		p < 0.05		n.s.	
−	+	+	%	PreW	44.5 ± 13.7	n.s.	43.5 ± 14.1	n.s.	40.7 ± 12.7	n.s.
				PostW	45.0 ± 13.2	p < 0.05	33.8 ± 10.5	n.s.	36.3 ± 12.2	n.s.
				P* (PreW × PostW)	n.s.		p < 0.05		n.s.	

Proportions of macrophages isolated from subcutaneous adipose tissues of women of premenopausal age (*SCAT*, n = 15) and from visceral and perivascular adipose tissues (*VAT* and *PVAT*, n = 16) of women of postmenopausal age (SCAT, n = 16, VAT and PVAT, n = 17), *n.s.* non-significant

The results are expressed as the mean of the proportion ±SD and by significance according to the Student’s paired parametric t test and the * Student’s unpaired parametric t test

Similarly, no differences in the presence of CD16+36^high^ macrophages in the different adipose tissues were found in women of premenopausal age. The lower proportion of these subpopulations in the SCAT compared to the VAT and PVAT that was observed in the entire group evaluation increased in the women of postmenopausal age (p < 0.0005 and p < 0.001, respectively). Although the proportions of CD16+ CD36^high^ macrophages were higher in the VAT and PVAT of women of postmenopausal compared to premenopausal age, these differences reached significance (p < 0.05) only in the PVAT.

Upon analyzing the M1 macrophage subpopulations, it was evident that although the lower proportions of CD16+36^high^163− macrophages in the SCAT compared to the VAT and PVAT were significant in both female groups, these differences in the subgroup of postmenopausal age women were very high (p < 0.0005). Additionally, only women of postmenopausal age displayed a slightly lower proportion of CD16+36^high^163− macrophages in the VAT compared to the PVAT (p < 0.05). Women of postmenopausal age had a higher proportion of CD16+36^high^163− macrophages in the VAT compared to women of premenopausal age (p < 0.05). However, no differences were found when the SCAT and PVAT were compared.

The women of postmenopausal age had a higher proportion of anti-inflammatory CD16− CD36^low^ CD163+ subpopulations in the SCAT compared to the VAT (p < 0.05). However, these differences were not detected in women of premenopausal age. Women of postmenopausal age also had a slightly lower proportion of these macrophages in the VAT (p < 0.05) compared to women of premenopausal age.

## Discussion

Our most interesting findings were as follows:

We confirmed that human adipose tissues contained both CD16 positive and CD16 negative macrophages. We demonstrated that the proportions of these subpopulations differed between the SCAT and VAT or PVAT. Our data also revealed that CD36 expression markedly differed between the CD16+ and CD16− macrophages. Although high CD36 expression (CD36^high^) was detected in the CD16+ macrophages, the CD16− macrophages did not exhibit CD36^high^ positivity. Conversely, the CD16− macrophages were primarily CD36^low^ and this phenotype was also uniformly CD163 positive.

The proportion of CD16+ CD36^high^ CD163− macrophages was doubled in the VAT and PVAT compared to the SCAT. It is tempting to speculate that this phenomenon reflects the higher metabolic and pro-inflammatory activities of visceral adipose tissues compared to subcutaneous tissues as has been repeatedly shown [26].

Although increased ATMs in obese adipose tissue have been repeatedly described, few studies have included quantification per gram of adipose tissue. Our results of total macrophage numbers per g of adipose tissue are in agreement with published studies [18, 19].

Others have also studied CD16 marker positivity in human subcutaneous adipose tissues. Kovacikova [18] found a slightly higher proportion of CD16 positive macrophages (approximately 60 %) compared to our data (Table 2), whereas other authors [4, 27] showed only a minor CD16 positive subpopulation in the same types of adipose tissue. Because no data on CD16 positive macrophages in visceral adipose tissues have been published, we cannot compare our results with others. In our study, ATMs showed a clearly different pattern when divided according to their CD16 expression levels when the CD36 and CD163 receptors were followed (Table 2). This finding indicates that the CD16 marker is not sufficient to fully distinguish pro-inflammatory macrophage subpopulations. A higher presence of CD16+ macrophages was found in the VAT compared to the SCAT (Table 2). This finding is compatible with the proposed higher importance of VAT in subclinical inflammation induction due to adipose tissue accumulation.

CD36 is one of the pattern recognition receptors and is expressed not only in monocytes/macrophages but also in adipocytes, platelets, and other cell types. CD36 is upregulated during the late stages of monocyte differentiation [28] and serves as a scavenger receptor that binds different ligands, including oxidized low-density lipoprotein, oxidized phospholipids, long chain acids, cell-derived microparticles, and apoptotic cells [29]. The interaction of CD36 with its ligands induces a monocyte influx into tissues and the secretion of pro-inflammatory cytokines [29]. CD36 is also a fatty acid translocase that is involved in fatty acid uptake and esterification in macrophages; thus, CD36 is regarded as important in foam cell formation during the progression of atherosclerosis [30]. The expression of the CD36 receptor in tissues can be modified by diet [31]. A very recent study identified a strong correlation between CD36 expression on ATMs and metabolic dysfunction [24]. Our data reveal that its expression differs markedly between CD16+ and CD16− macrophages. In our study, CD16+ macrophages possessed both high (CD36^high^) and low (or CD36^low^) CD36 expression, whereas CD16− macrophages were not ever CD36^high^ positive and expressed a low level of CD36 (CD36^low^). We hypothesize that the differences in CD36 expression clearly confirm that we have applied an appropriate method to delineate the CD16+ and CD16− macrophage subpopulations (Fig. 1).

The hemoglobin scavenger receptor CD163 is exclusively expressed on monocytes/macrophages. Its expression in ATMs differs according to their location (omental vs. subcutaneous) [19, 20]. CD163 is an M2 marker of alternatively stimulated macrophages in in vitro studies [32–34], and the CD163 phenotype has also shown to be more frequent in subcutaneous ATMs after weight loss in men [20]. Contrary to the pro-inflammatory macrophages, CD163 expression is reported to suppress the immune mechanism through local interleukin-10 secretion [35]. Additionally, recently published nomenclature guidelines categorize CD163 as a marker of anti-inflammatory M2 macrophages [12].

We demonstrated (Table 2) that all CD16− macrophages were CD163 positive in all three types of adipose tissue. Therefore, we believe that the CD16− CD36^low^ CD163+ population should correspond to anti-inflammatory non-classical macrophages. The situation with CD16+ macrophages is different because the great majority of CD16+ CD36^high^ macrophages in the VAT and PVAT do not express substantial levels of CD163. However, recent results have shown [24] that stimulation of macrophages in adipose tissue does not follow the classical activation pathway and that CD36 expression in macrophages can increase under activation in a metabolically unhealthy environment (i.e., high glucose, insulin and palmitate concentrations). This environment leads to metabolic activation that is independent of the pro-inflammatory pathways. Therefore, these macrophages should be considered metabolically activated pro-inflammatory macrophages. Although it is difficult to establish an additional value of the CD163 marker for the M1/M2 dichotomy, its role is plausible in connection with the above-mentioned data. In our view, the fact that a large majority (95 %) of CD16− macrophages were also CD163+ strongly supported their classification as M2 macrophages.

We also found co-expression of pro- and anti-inflammatory markers of ATMs (i.e., CD16+ CD36^high^ CD163+ macrophages), which seemed to confirm the prevailing opinion that changes in the local microenvironment in situ might evoke varying mixtures of M1 and M2 responses, resulting in overlaps and a broad spectrum between the M1 and M2 macrophage phenotypes [20, 36, 37]. Interestingly, this phenotype was slightly higher in the SCAT compared to the other adipose tissues.

Our group of living kidney donors represents apparently healthy individuals. Their BMIs, prevalence of hypertension, and dyslipoproteinemia were slightly lower compared to the last population survey of a representative Czech population sample reported by the post-MONICA WHO project of 2007 [38]. Additionally, the cholesterol and non-HDL cholesterol levels in our group were approximately 17 % lower than the general population. Nevertheless, the exclusion criteria for living kidney donors have slightly shifted in our transplantation center similar to other centers, and three hypertonic individuals with dyslipoproteinemia and seven obese individuals were accepted into the program.

Based on the results shown in Tables 2 and 3, several points should be highlighted. First, the inclusion of two additional macrophage markers (CD36 and CD163) improves the M1 and M2 definition compared to the use of only one of these markers. Second, the VAT and PVAT display a higher pro-inflammatory M1 proportion than the SCAT, whereas the situation with M2 is obviously reversed. Third, there are no important differences in the ATM subpopulations when all females are compared to males, but significant differences are detectable between pre- and postmenopausal women. All pro-inflammatory parameters are increased upon the menopausal change; this shift highlights the importance of this period during cardiovascular risk changes.

The limitations of the present study are primarily the relatively low number of individuals included in the study and our strict definitions of the M1 and M2 subpopulations, which were based on our interpretation of the available information from the literature and our own data.

## Conclusions

Using a unique approach, we were able to identify macrophage phenotypes present in human adipose tissues and to compare subcutaneous with visceral and perivascular fat in identical subjects. Nevertheless, it is still too early to determine how the differences in macrophage subpopulations reflect the physiological functions of adipose tissues or how they affect interactions between adipose tissue cells.

## Abbreviations

SCAT: subcutaneous adipose tissue
VAT: visceral adipose tissue
PVAT: perivascular adipose tissue
ATM: adipose tissue macrophages
SVF: stromal vascular fraction
BMI: body mass index
hsCRP: high-sensitivity C-reactive protein
preW: women of premenopausal age
postW: women of postmenopausal age
